# Supplementary material for: Spatially-resolved transcriptomics reveal macrophage heterogeneity and prognostic significance in diffuse large B-cell lymphoma
Source: Nat Commun. 2024 Mar 8;15:2113. doi: 10.1038/s41467-024-46220-z (PMC10923916; doi:10.1038/s41467-024-46220-z)
Supplement: Supplementary file 1 — Supplementary information [file 41467_2024_46220_MOESM1_ESM.pdf]

Supplementary Appendix: Spatially-resolved transcriptomics reveal macrophage heterogeneity and prognostic significance in diffuse large B-cell lymphoma

Contents

Investigators 1

Supplementary Methods 4

Supplementary Figures..6

Supplementary Tables 21

Supplementary References...26

Investigators

Authors: Min Liu[1,2,3]; Giorgio Bertolazzi[4,5]; Shruti Sridhar[1]; Rui Xue Lee[1]; Patrick Jaynes[1]; Kevin Mulder[6,7,8]; Nicholas Syn[9,10]; Michal Marek Hoppe[1]; Fan Shuangyi[9]; Yanfen Peng[1]; Jocelyn Thng[1]; Reiya Chua[11]; Jayalakshmi[11]; Yogeshini Batumalai[11]; Sanjay De Mel[11,12], Limei Poon[11,12]; Esther Chan[11,12]; Joanne Lee[11,12]; Susan Swee-Shan Hue[9,12]; Sheng-Tsung Chang[13]; Shih-Sung Chuang[13]; K George Chandy[14]; Xiaofei Ye[15]; Qiang Pan-Hammarström[16]; Florent Ginhoux[6,7,8]; Yen Lin Chee[11,12]; Siok-Bian Ng[1,9,12]; Claudio Tripodo[5,17*]; Anand D. Jeyasekharan[1,11,12,18*]

Affiliations:

[1]Cancer Science Institute of Singapore, National University of Singapore, Singapore

[2]Department of Radiation Oncology, Chongqing University Cancer Hospital, Chongqing, P. R. China

[3]Department of Immunology, Tianjin Medical University Cancer Institute and Hospital, Tianjin, P. R. China

[4]Department of Economics, Business and Statistics, University of Palermo, Palermo, Italy.

[5]Tumor Immunology Unit, Department of Sciences for Health Promotion and Mother-Child Care "G. D'Alessandro", University of Palermo, Palermo, Italy

[6]Singapore Immunology Network, Agency for Science, Technology and Research, Singapore

[7]Institut National de la Santé Et de la Recherche Medicale (INSERM) U1015, Equipe Labellisée—Ligue Nationale contre le Cancer, 94800 Villejuif, France

[8]Université Paris-Saclay, Gustave Roussy, Villejuif, France

[9]Department of Pathology, Yong Loo Lin School of Medicine, National University of Singapore, Singapore

[10]Department of Biomedical Informatics, Yong Loo Lin School of Medicine, National University of Singapore, Singapore

[11]Department of Haematology-Oncology, National University Health System, Singapore

[12]NUS Centre for Cancer Research, Yong Loo Lin School of Medicine, National University of Singapore, Singapore

[13]Department of Pathology, Chi-Mei Medical Center, Tainan City, Taiwan

[14]Lee Kong Chian School of Medicine, Nanyang Technological University Singapore, Singapore

[15]Kindstar Global Precision Medicine Institute, Wuhan, China

[16]Division of Immunology, Department of Medical Biochemistry and Biophysics, Karolinska Institutet, Stockholm, Sweden

[17] Histopathology Unit, Institute of Molecular Oncology Foundation (IFOM) ETS - The AIRC Institute of Molecular Oncology, Milan, Italy

[18]Department of Medicine, Yong Loo Lin School of Medicine, National University of Singapore, Singapore

Supplementary Methods

Immunofluorescence staining

The formalin-fixed paraffin-embedded sections (3 μm thick) from CMMC DLBCL cohort (n = 86) were baked at 60°C for 20 min, and loaded into the slide tray on Bond Max (Leica Biosystems) for deparaffinization, rehydration, antigen retrieval. Subsequently, the slides were incubated with primary antibody (C1Q antibody, Ab268120, Abcam, Cambridge, UK) for 20 min, incubated with anti-mouse IgG HRP secondary antibody for 10 min, and incubated Opal TSA staining for 5 min, followed by a full cycle starting from antigen retrieval to stain with another primary antibody (CD68 antibody, M0876, Dako, California, USA). Finally, slides were mounted with DAPI and mounting medium. Images were acquired using the Vectra 2 imager and analysed using inForm 2.6.0 (Akoya Biosciences, Massachusetts, USA). Cells were segmented with DAPI nuclear staining. The mean membrane intensity and mean cytoplasm intensity per cell were captured for CD68 and C1Q, respectively. For each image, cells were deemed as positive (phenotyped) for CD68 through an algorithm within inForm, based on factors such as localized background signals and morphology. As C1Q shows a wide range of expression levels within CD68 cells, no specific cut-off for positivity could be assigned. Therefore, for each patient, the mean pixel intensity of C1Q per CD68+ cell was measured, and the cohort was divided across the median to compare C1Q high vs low cases. Based on this score, a Kaplan-Meier analysis was performed to estimate the survival association between high and low groups, stratified by the median intensity of C1Q in CD68+ cells. The log-rank test was used to test the

differences in the OS between these two groups.

Supplementary Figures

Supplementary Fig. 1

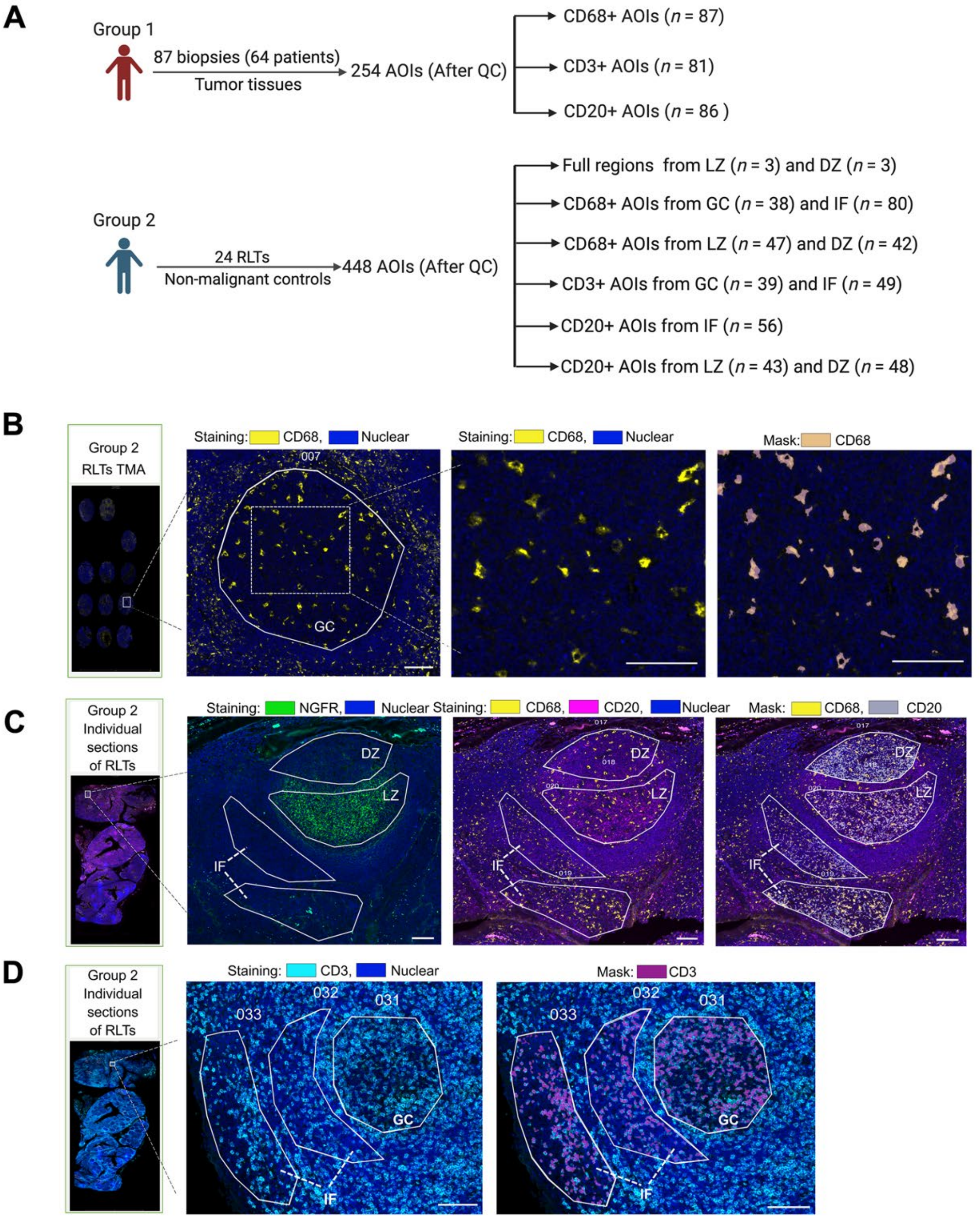

Supplementary Fig. 1. DSP ROI collection summary on the study population, and immunofluorescence staining in different types of segmentation. (A) Schematic of the study population and the corresponding numbers of DSP AOIs collected (created with BioRender.com). **(B)** For the RLT TMA, CD68 and SYTO 13 were used to respectively stain macrophages (yellow) and nuclei (blue). GC macrophages were collected based on the staining masks generated on GeoMx. Source data are provided as a Source Data file. **(C)** For DSP of individual tonsil sections, serial sections were used. To distinguish the regions of the GC, the first section was stained with NGFR to illuminate the LZ (green). The subsequent section was stained with macrophages were stained with CD68 (yellow) , B cells were stained with CD20 (magenta) and nuclei were stained with SYTO 13 (blue). Macrophages and B cells of LZ, DZ, and IF were collected respectively based on their corresponding staining masks. Source data are provided as a Source Data file. **(D)** CD3 stained T cells (cyan), and SYTO 13 stained nuclei (blue). T cells of GC and IF were collected based on the staining masks of CD3. Representative images were shown. Scale bar: 100 μm. Source data are provided as a Source Data file. Digital spatial profiling, DSP; reactive lymphoid tissue, RLT; tissue microarray, TMA; regions of interest, ROI; areas of interest, AOI; nerve growth factor receptor, NGFR; light zone, LZ; dark zone, DZ; interfollicular, IF, germinal center, GC.

Supplementary Fig. 2

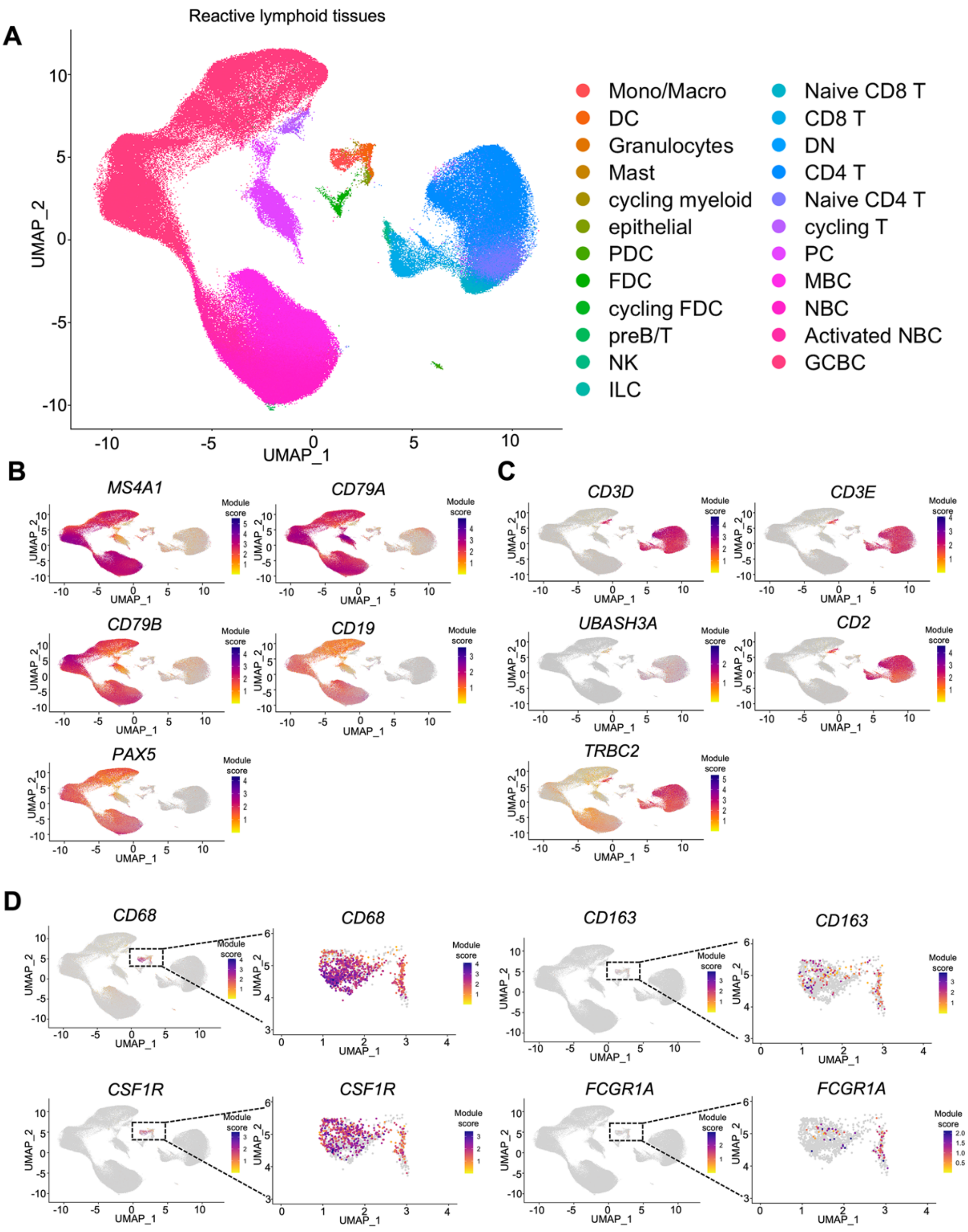

Supplementary Fig. 2. Signature validation of B cells, T cells, and macrophages on scRNA-seq datasets of RLTs. (A) UMAP of the HCA tonsil dataset containing an integrative single cell atlas of over 209,786 cells, with a glossary of annotated cell types. **(B-D)** Expression of individual genes representing B cells (*MS4A1*, *CD79A*, *CD79B*, *CD19*, and *PAX5*), T cells (*CD3D*, *CD3E*, *UBASH3A*, *CD2*, and *TRBC2*), and macrophages (*CD68*, *CD163*, *CSF1R*, and *FCGR1A*) were projected onto the scRNA-seq atlas to assess enrichment. Single cell sequencing, scRNA-seq; Uniform Manifold Approximation and Projection, UMAP; activated naive B cells, NBC; GC B cells, GCBC; plasma cells, PC; memory B cells, MBC; Double negative T cells, DN; Innate Lymphoid cells, ILC; Natural Killer cells, NK; Precursor B/T cells, pre B/T; follicular dendritic cells, FDC; Monocytes/Macrophages, Mono/Macro.

Supplementary Fig. 3

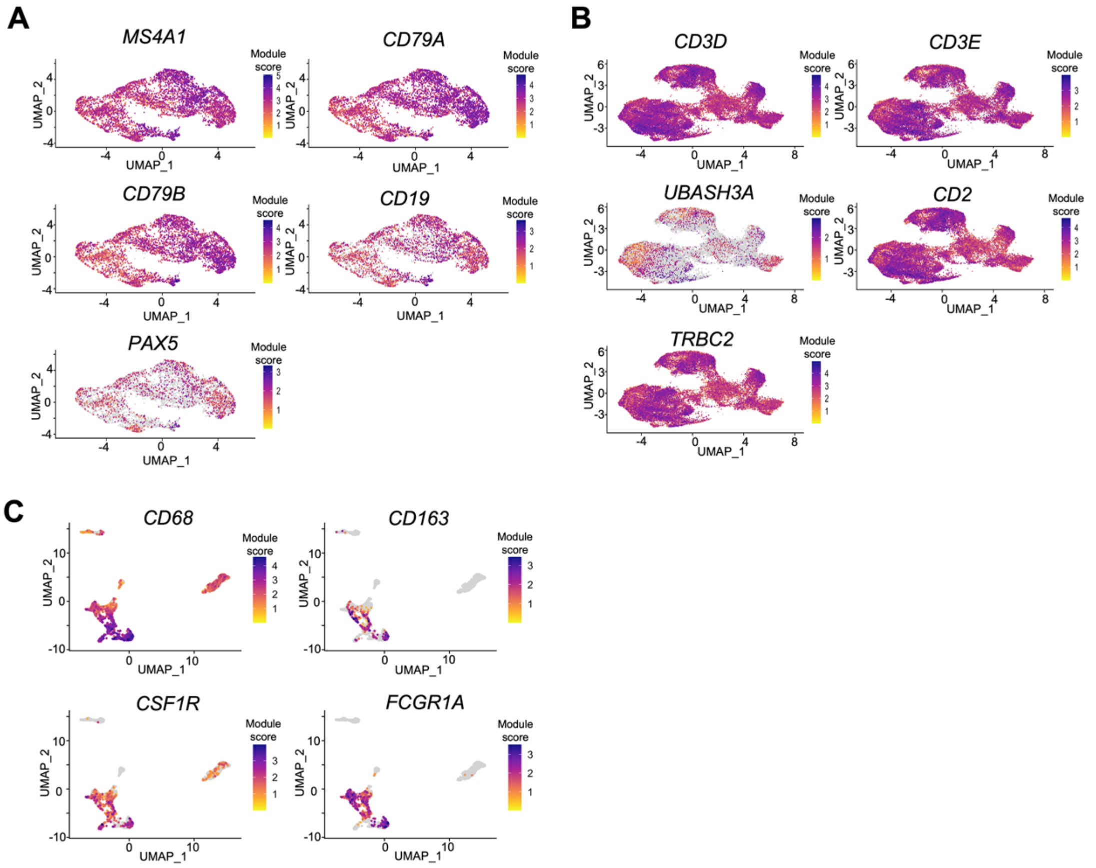

Supplementary Fig. 3. Signature validation of B cells, T cells, and macrophages on scRNA-seq datasets of DLBCL tissues. (A-C) From the scRNA-seq dataset of DLBCL produced by Ye et al, expression of individual genes representing B cells (*MS4A1*, *CD79A*, *CD79B*, *CD19*, and *PAX5*), T cells (*CD3D*, *CD3E*, *UBASH3A*, CD2, and *TRBC2*), and macrophages (*CD68*, *CD163*, *CSF1R*, and *FCGR1A*) were projected onto the scRNA-seq atlas to assess enrichment. Original UMAPs with annotations for non-malignant B-cells, T-cells and myeloid cells can be found in Ye et al [1].

Supplementary Fig. 4

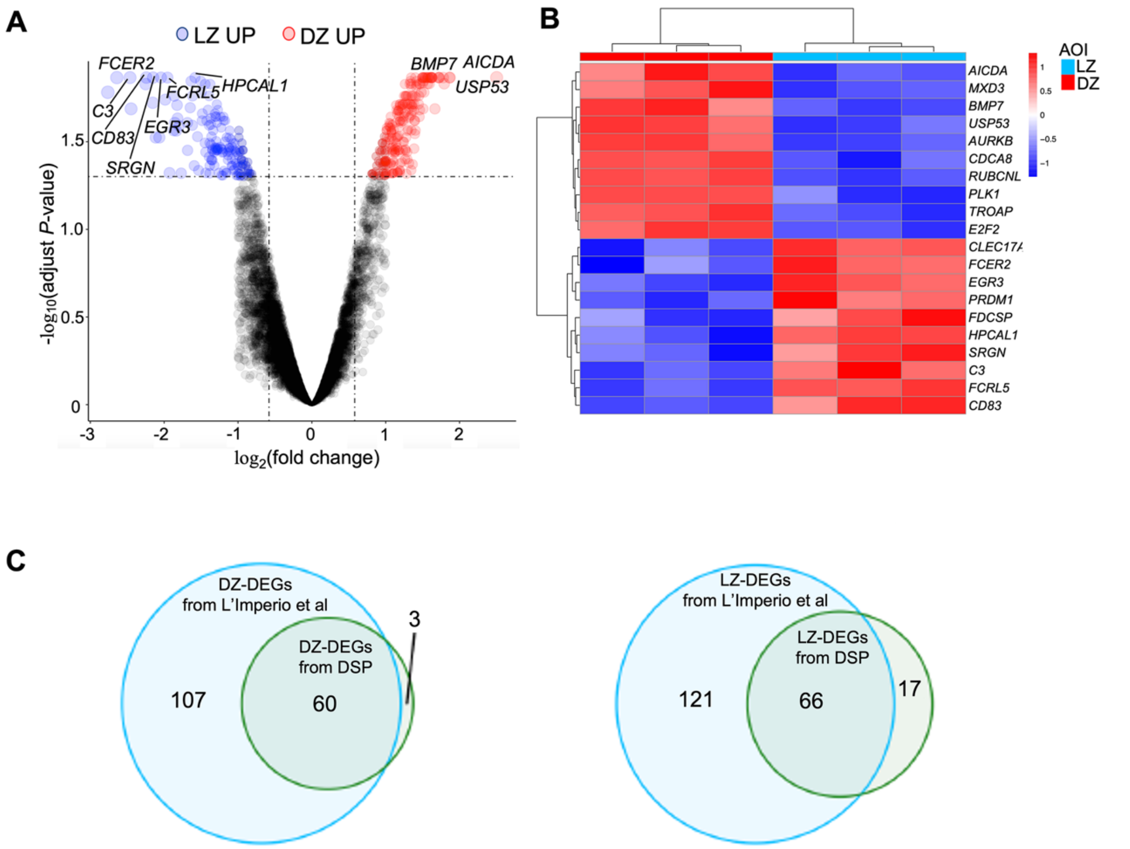

Supplementary Fig. 4. Validation of gene expression patterns in the LZ and DZ **(A)** Volcano plot showing the DEGs of full regions (all cells, majority CD20+) between LZ and DZ (n = 6) based on adjusted P value < 0.05 and |$\log_2$FC| ≥ 0.58). P values were determined by two tailed moderated t-test (BH corrected). **(B)** Top DEGs (10 DEGs upregulated in LZ and 10 DEGs upregulated in DZ) based on adjusted P value are displayed in the heatmap. **(C)** Venn diagram displaying the overlapping DEGs from full regions of LZ and DZ between our DSP data and the previous publication from L'Imperio. Differentially expressed genes, DEGs.

Supplementary Fig. 5

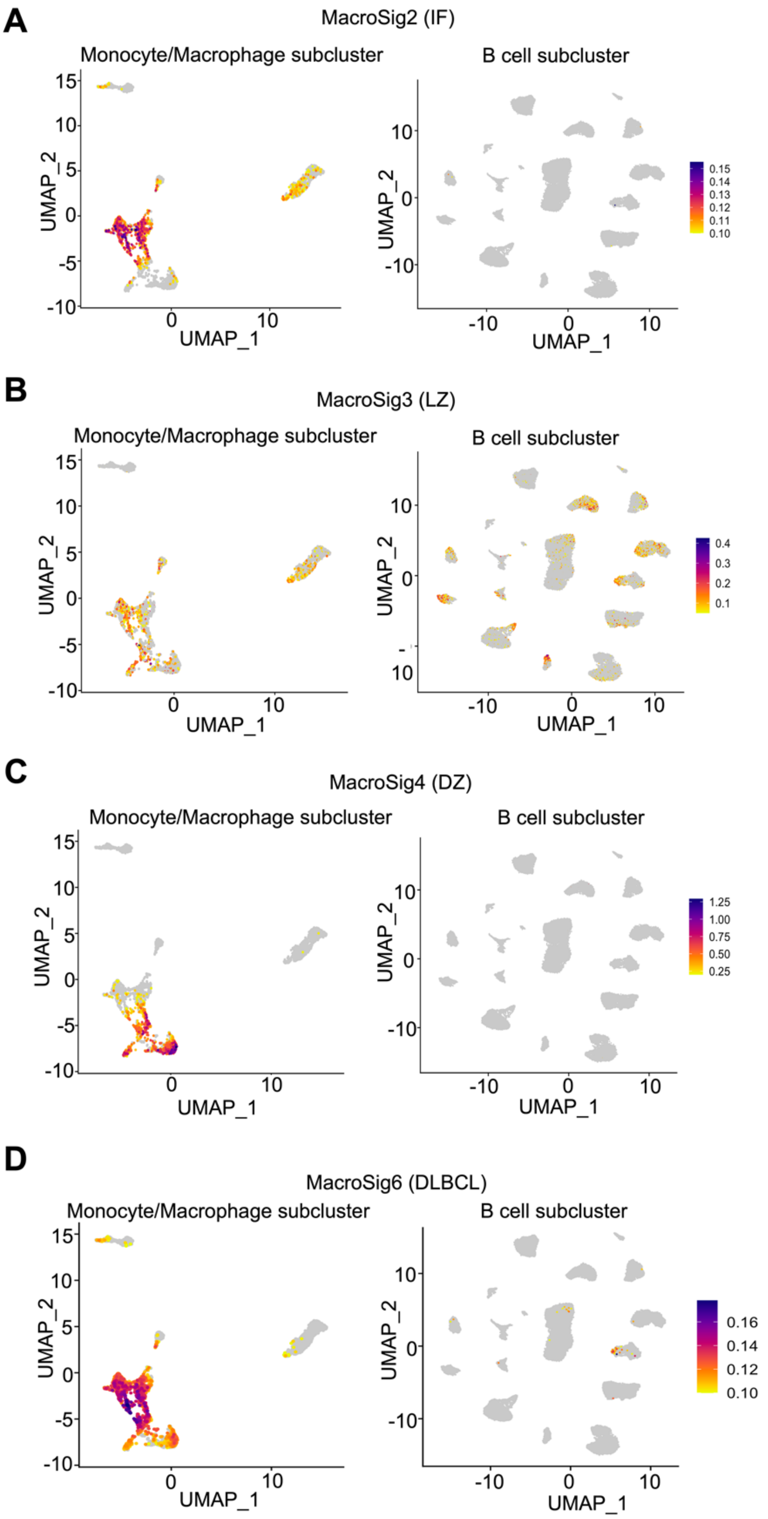

Supplementary Fig. 5. Validating MacroSig2,3,4,6 using DLBCL scRNA-seq datasets. (A-D) All genes of MacroSig2,3,4,6, through their respective module scores, were projected onto the Monocyte/Macrophage and B cell subsets of DLBCL scRNA-seq datasets (Ye et al; n = 17).

Supplementary Fig. 6

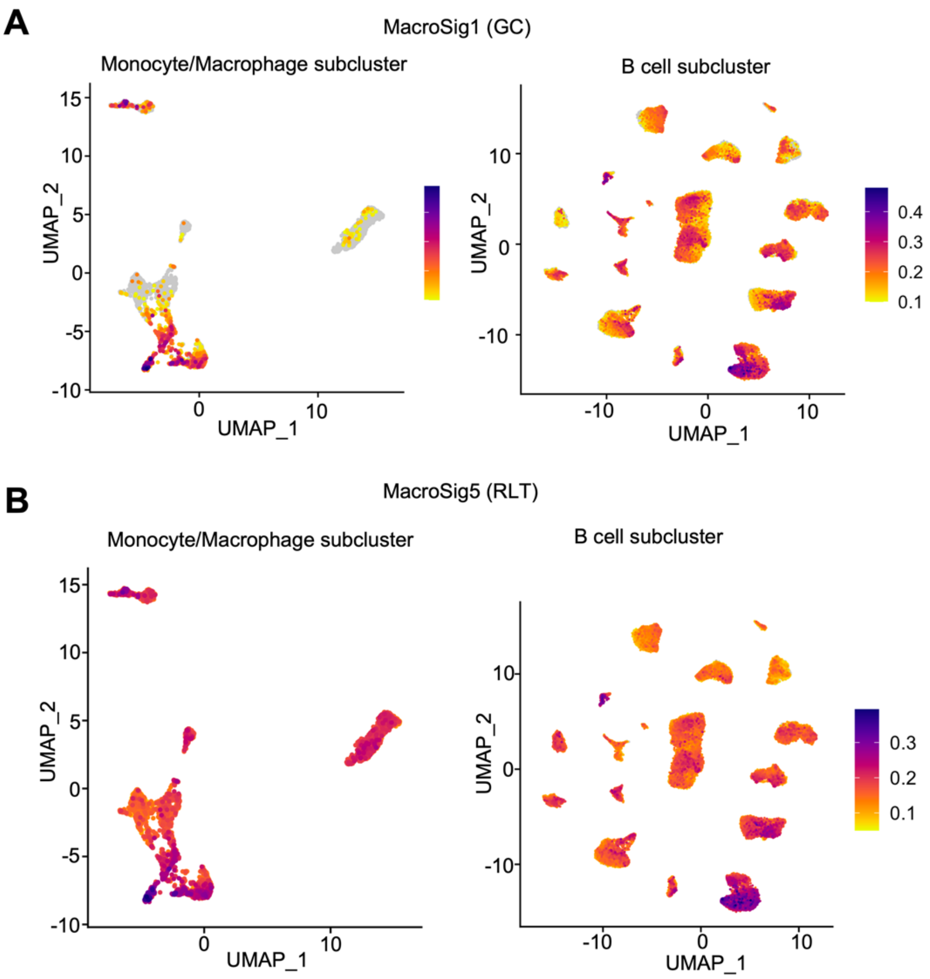

Supplementary Fig. 6. Validating MacroSig1, 5 using DLBCL scRNA-seq datasets. (A-B) All genes of MacroSig1,5 through their respective module scores, were projected onto the Monocyte/Macrophage and B cell subsets of DLBCL scRNA-seq datasets (Ye et al; *n* = 17).

Supplementary Fig. 7

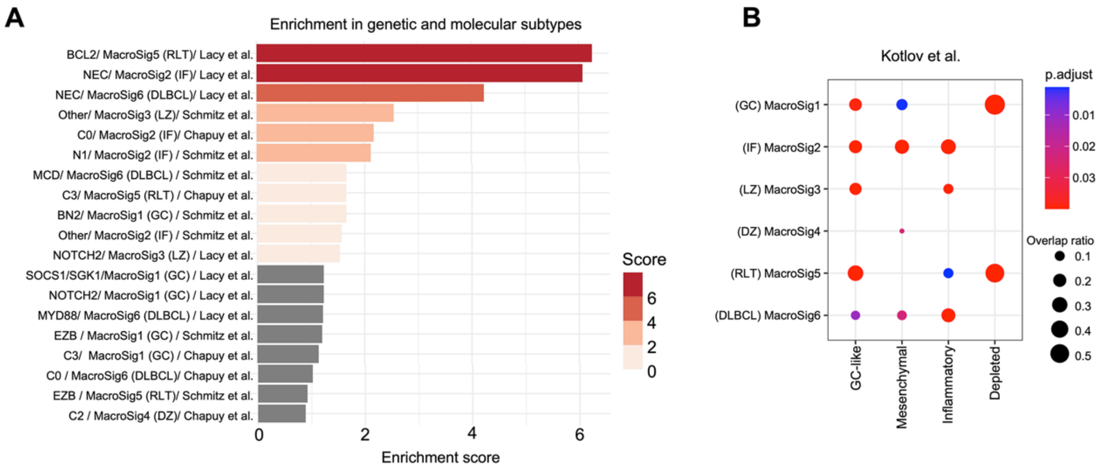

Supplementary Fig. 7. The relationship of spatially-derived MacroSigs with established genetic DLBCL subclassifications. (A) Enrichment analysis of all MacroSigs was performed on the genetic and molecular DLBCL subtypes across three distinct bulk RNA gene expression profiles of Schmitz et al., Lacy et al., and Chaupy et al. as mentioned in the Methods. The genetic subtypes association analysis is presented as an integrated bar graph, where the strength of association between MacroSigs and genetic subtypes is represented by an enrichment score calculated by: -log10 (adjusted Fisher *P* value) **(B)** Enrichment gene set analysis of all MacroSigs was performed on DLBCL microenvironment categories generated by Kotlov et al. Count refers to the number genes present in the overlap between the MacroSigs and DLBCL microenvironment categories. The overlap ratios were obtained by dividing the count by the total number of genes in that respective DLBCL microenvironment categories. *P* value generated by Fisher exact test (BH corrected). Not elsewhere classified, NEC.

Supplementary Fig. 8

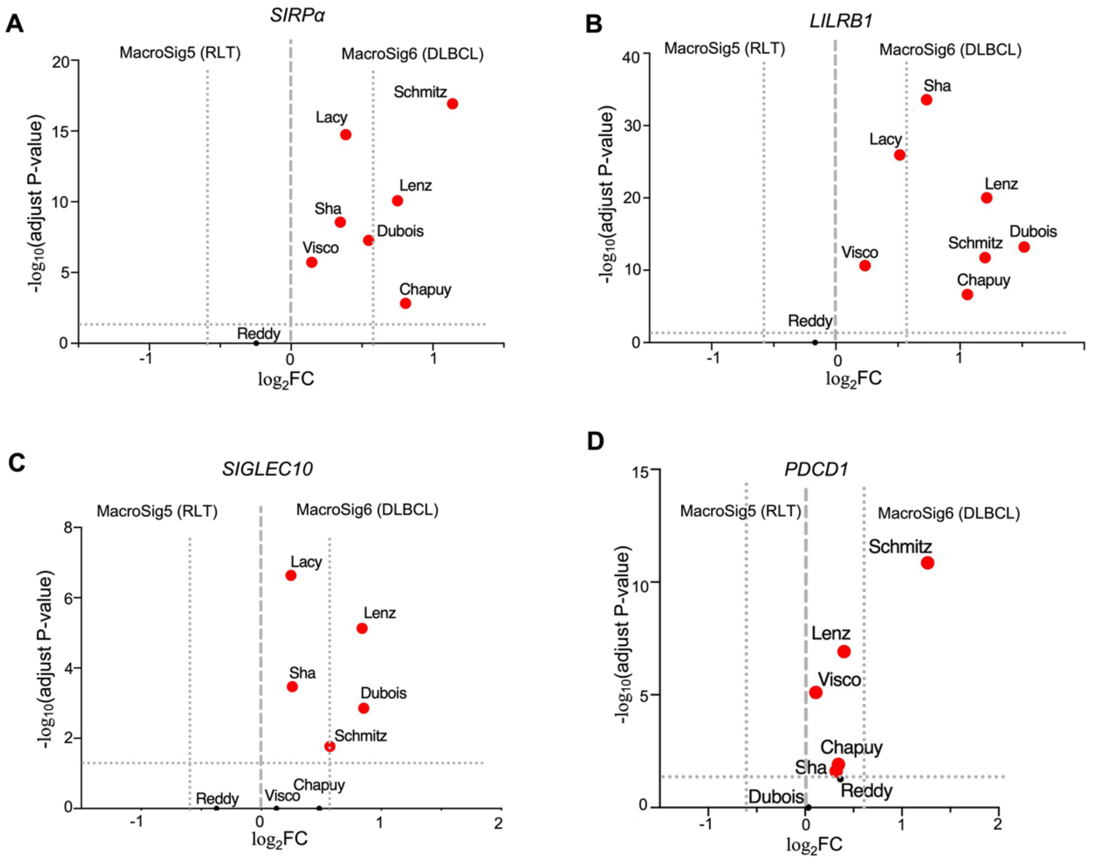

Supplementary Fig. 8. Macrophage checkpoints are enriched in patients categorized to have MacroSig 6 (DLBCL) in comparison to MacroSig 5 (RLT). **(A-D)** Using eight DLBCL publicly available datasets (n = 4, 594; Lenz et al, Visco et al, Dubois et al, Reddy et al, Chapuy et al, Reddy et al, Sha et al, and Lacy et al), patients were divided into high and low groups based on the expression levels of MacroSig5/6 in each dataset (see Methods: Survival Analysis for details on patient stratification). Differential expression analyses were performed to evaluate the average expression of macrophage phagocytosis checkpoints (*SIRPα*, *LILRB1*, *SIGLEC10*, and *PDCD1*) between two groups using the Wilcoxon test. *P* values were adjusted using the Bonferroni correction. *PDCD1* was not present in Lacy dataset.

Supplementary Fig. 9

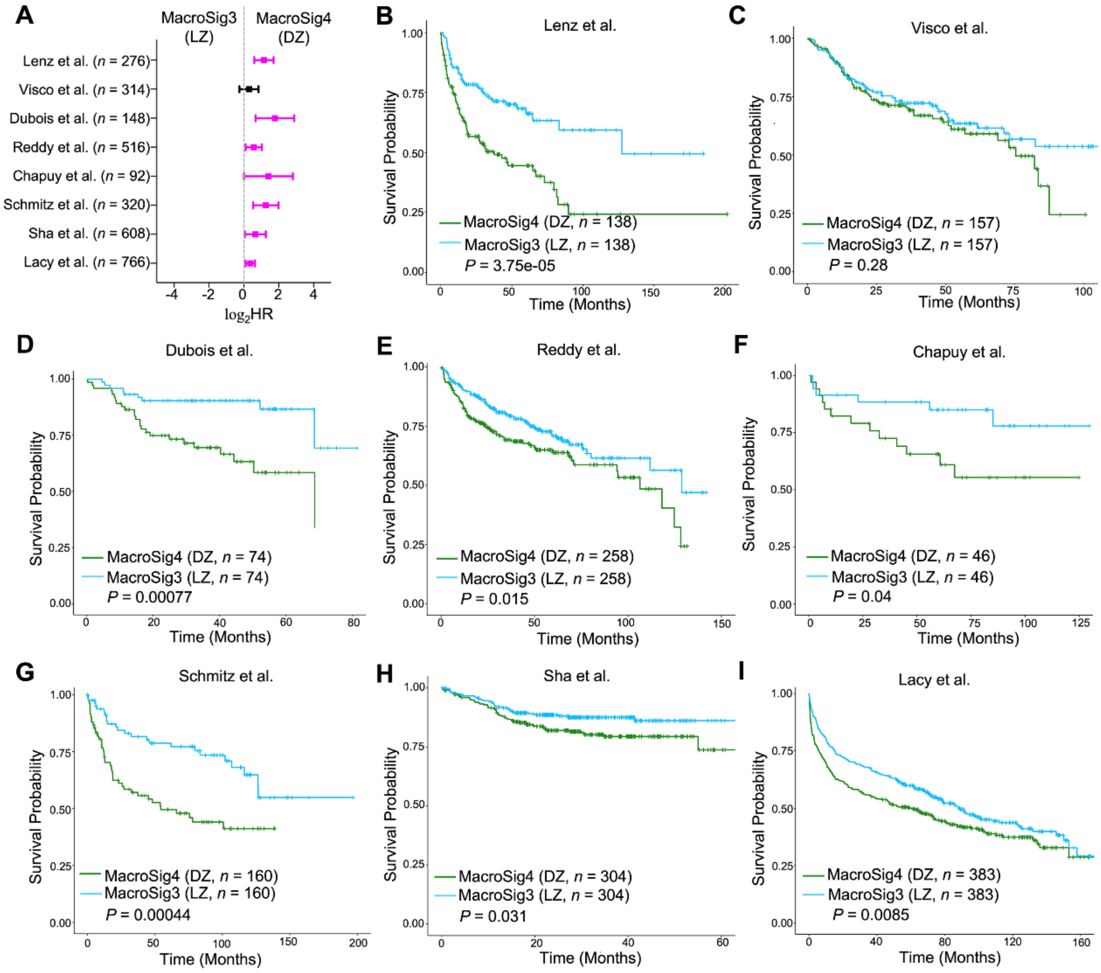

Supplementary Fig. 9. Spatially-derived MacroSig3/4 (LZ/DZ) after filtering transcripts potentially linked to close interactions between macrophages and T cells, obtained analogous results in terms of prognostic ability. (A) Forest plot depicting the univariate Cox proportional hazards model analysis, comparing MacroSig3 (LZ) and MacroSig4 (DZ) after filtering transcripts potentially linked to close interactions between macrophages and T cells (represented as tertile groups, as described in Methods: Survival analysis). Analysis was applied to bulk RNA gene expression profiles of DLBCL patients across eight publicly available transcriptomic datasets (n = 4,594, 8 datasets). Data are presented as the 95% confidence interval of the hazard ratio (plotted in log-scale). Source data are provided as a Source Data file. **(B-I)** Kaplan–Meier analyses showed that patients with high expression of MacroSig4 (DZ) and low expression of MacroSig3 (LZ) after filtering transcripts potentially linked to close interactions between macrophages and T cells were still associated with poor OS in DLBCL patients across seven distinct DLBCL datasets. P values generated by log-rank test.

Supplementary Fig. 10

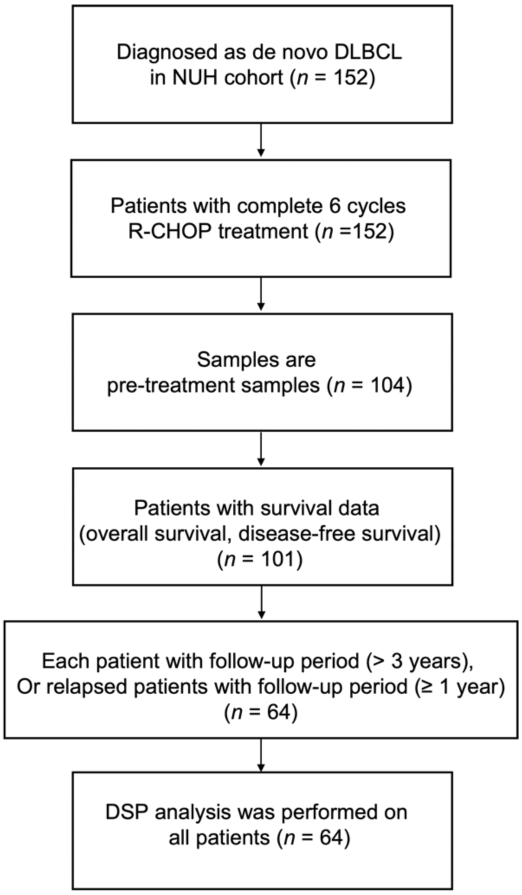

Supplementary Fig. 10. Patients' selection flowchart. Cases of de novo DLBCL diagnosed between 2010 and 2017 at the National University Hospital Singapore were included in this study. The criteria for selecting patients for DSP analysis is shown.

Supplementary Fig. 11

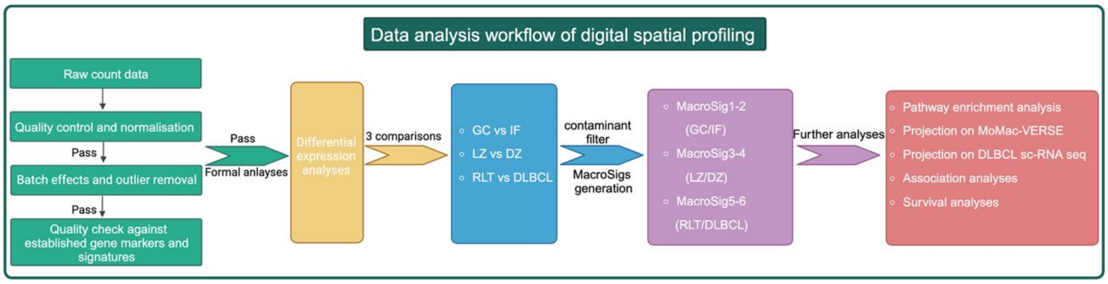

Supplementary Fig. 11. The analysis workflow of DSP data. Raw count data was subjected to quality checks and validations before undergoing formal differential expression analyses. Six MacroSigs were eventually generated through DEG comparisons between GC/IF, LZ/DZ and RLT/DLBCL. The MacroSigs were then used for further analyses such as macrophage subtype identification, pathway enrichment/association analyses, and survival analyses (created with BioRender.com).

Supplementary Tables

Supplementary Table 1

Supplementary Table 1. Gene signature characteristics of Macrophages, T cells, and B cells

Macrophages	T cells	B cells
CD68	CD3D	MS4A1
CD163	CD3E	CD79A
FCGR1A	UBASH3A	CD79B
CSF1R	CD2	CD19
	TRBC2	PAX5

Supplementary Table 2

Supplementary Table 2. HR and 95% CI of overall survival in DLBCL patients after 6 cycles of R-CHOP

Groups	Datasets	HR	95% CI	*P* value
MacroSig6 (DLBCL) versus MacroSig5 (RLT)	Lenz et al. (*n* = 420)	2.5	1.68-3.71	5.6e-06
	Visco et al. (*n* = 498)	1.66	1.13-2.44	0.00968
	Dubois et al. (*n* = 223)	2.96	1.31-6.69	0.00903
	Reddy et al. (*n* = 773)	1.42	1.03-1.95	0.0345
	Chapuy et al. (*n* = 137)	1.35	0.49-3.72	0.565
	Schmitz et al. (*n* = 481)	1.82	1.09-3.01	0.0210
	Sha et al. (*n* = 913)	1.07	0.72-1.57	0.7406
	Lacy et al. (*n* = 1,149)	1.29	1.06-1.57	0.00968
MacroSig4 (DZ) versus MacroSig4 (LZ)	Lenz et al. (*n* = 420)	3.25	2.16-4.9	1.4e-08
	Visco et al. (*n* = 498)	1.39	0.96-2	0.0792
	Dubois et al. (*n* = 223)	4.3	1.86-9.95	0.000662
	Reddy et al. (*n* = 773)	1.81	1.32-2.47	0.000201
	Chapuy et al. (*n* = 137)	3.39	1.41-8.15	0.00631
	Schmitz et al. (*n* = 481)	1.84	1.12-3.02	0.0161
	Sha et al. (*n* = 913)	2.36	1.56-3.59	5.3e-05
	Lacy et al. (*n* = 1,149)	1.79	1.48-2.17	2.2e-06

Cox proportional hazards model and the Kapan-Meier method were used for analysis. Before fitting the Cox model and conducting the log-rank test, the cox.ph test was used to test the proportional hazard assumption. *P* values were determined by two tailed. Diffuse large B-cell lymphoma, DLBCL; rituximab with cyclophosphamide, doxorubicin, vincristine, and prednisolone (R-CHOP); macrophage signatures, MacroSigs; reactive lymphoid tissue, RLT; confidence interval, CI; light zone, LZ; dark zone, DZ.

Supplementary Table 3

Supplementary Table 3. Multivariate analysis of MacroSig4 (DZ) adjusted for IPI scores and DHL*

Dataset	Groups	Adjust factors	OS		
			HR	95% CI	*P* value
Dubois (*n* = 223)	MacroSig4 (DZ) versus MacroSig4 (LZ)	IPI	3.48	1.49-8.12	0.0039
Reddy (*n* = 773)	MacroSig4 (DZ) versus MacroSig4 (LZ)	IPI and DHL (negative, positive)	2	1.1-3.64	0.023
Chapuy (*n* = 137)	MacroSig4 (DZ) versus MacroSig4 (LZ)	IPI	2.65	1.08-6.51	0.034
Schmitz (*n* =481)	MacroSig4 (DZ) versus MacroSig4 (LZ)	IPI	1.52	0.85-2.72	0.16
Sha (*n* = 913)	MacroSig4 (DZ) versus MacroSig4 (LZ)	IPI, DHL (double-hit, MYC-normal, single-hit)	2.38	1.23-4.61	0.01
Lacy (*n* = 1149)	MacroSig4 (DZ) versus MacroSig4 (LZ)	IPI	1.58	1.23-2.04	0.0004

*The IPI scores of Lenz et al. and Visco et al. were not available. The DHL is only available in Reddy et al. and Sha et al. Cox proportional hazards model and the Kapan-Meier method were used for analysis. Before fitting the Cox model and conducting the log-rank test, the cox.ph test was used to test the proportional hazard assumption. *P* values were determined by two tailed. Dark zone, DZ; international prognostic index, IPI; double hit lymphoma, DHL; overall survival, OS; hazard ratio, HR; confidence interval, CI.

Supplementary Table 4

Supplementary Table 4. The gene lists of MacroSig3-4 (LZ/DZ) after filtering transcripts potentially linked to close interactions between macrophages and T cells

DZ vs LZ							
MacroSig3 (LZ)				MacroSig4 (DZ)			
Ranking	Gene	Log_2FC	Adjusted *P* value	Ranking	Gene	Log_2FC	Adjusted *P* value
1	CXCL13	2.31	8.1E-15	1	C1QA	2.02	7.4E-13
2	CLU	1.24	3.3E-12	2	CD163L1	1.68	1.7E-12
3	CHI3L1	1.51	0.000000011	3	C1QC	2.01	1E-10
4	CSTA	0.69	0.00000021	4	A2M	1.21	6.7E-10
5	CDC42EP4	0.92	0.00000043	5	C1QB	1.39	1.1E-09
6	ZBED6CL	0.82	0.00000081	6	SLC40A1	1.18	1.2E-09
7	PLEK	0.59	0.000035	7	LIPA	1.12	0.000000023
8	CHIT1	1.14	0.000035	8	CD163	1.4	0.000000067
9	TNFRSF9	0.7	0.000056	9	LGMN	1.48	0.00000017
10	MYC	0.69	0.00014	10	C3AR1	0.77	0.0000011
11	FPR1	0.71	0.00036	11	MS4A6A	0.82	0.0000011
12	ALPK2	0.66	0.00042	12	CTSB	0.85	0.0000011
13	SOCS3	0.76	0.00063	13	PDK4	1.11	0.0000022
14	DSP	0.81	0.00072	14	CCL18	1.22	0.0000034
15	NDRG1	0.71	0.00077	15	SDC3	0.84	0.000011
16	CHRNA1	0.64	0.001	16	IL18BP	0.89	0.000016
17	CYP27B1	0.84	0.0018	17	MPEG1	0.96	0.000018
18	HAMP	0.68	0.0021	18	CTSZ	0.67	0.000028
19	RAB13	0.61	0.0023	19	FCGRT	0.89	0.000036
20	NFKBIA	0.61	0.0038	20	PLA2G15	0.85	0.000041
21	GFPT2	0.68	0.0044	21	ABCA1	0.76	0.00012
22	DERL3	0.62	0.0057	22	PLD3	0.72	0.00013
23	GJA1	0.67	0.006	23	CFD	0.6	0.00014
24	IGHG2	0.71	0.007	24	ADORA3	0.72	0.00021
25	IGHG3	0.76	0.0084	25	SLC18B1	0.79	0.00032
26	TMEM178B	0.59	0.01	26	BCR	0.64	0.00035
27	IGHG1	0.78	0.013	27	FPR3	0.72	0.00043
28	JCHAIN	0.8	0.017	28	SELENOP	0.88	0.00054
29	VPS37B	0.66	0.017	29	ADAP2	0.67	0.00098
30	LGI2	0.62	0.02	30	RNASE6	0.66	0.001
31	NRG2	0.63	0.044	31	SLC35E3	0.71	0.0011
				32	FOXP1	0.59	0.0012
				33	CST3	0.65	0.0013
				34	CD209	0.87	0.0016
				35	PECAM1	0.62	0.002
				36	PYGL	0.69	0.0021
				37	CD300A	0.64	0.0022
				38	COL6A1	0.63	0.0032
				39	SLC37A2	0.64	0.0036
				40	PLTP	0.6	0.0038
				41	MCOLN1	0.62	0.004
				42	HPSE	0.77	0.005
				43	SLC7A8	0.77	0.006
				44	DHRS3	0.61	0.0064
				45	CCR1	0.58	0.0091
				46	COLEC12	0.58	0.0095
				47	LYZ	0.66	0.012
				48	MS4A4A	0.74	0.012
				49	IGFBP5	0.8	0.012
				50	ITM2B	0.65	0.014
				51	BLVRB	0.59	0.014
				52	NRP1	0.63	0.016
				53	ATP6V0D2	0.74	0.017
				54	BGN	0.67	0.018
				55	CASS4	0.62	0.022
				56	PILRA	0.59	0.032
				57	MMP12	0.78	0.033
				58	MAMDC2	0.61	0.037

Differential expressed genes, DEGs; light zone, LZ; dark zone, DZ; fold change, FC. *P* values were determined by two tailed moderated *t*-test (BH corrected). Genes ranked by *P* value.

Supplementary Table 5

Supplementary Table 5. The clinicopathologic characteristics in DLBCL patients from NUH cohort

Variables	N	Percentage
Age		
≤60y	26	40.6%
＞60y	38	59.4%
Sex		
Male	42	65.6%
Female	22	34.4%
Cell of origin		
Non-GCB	32	50.0%
GCB	31	48.4%
Undetermined	1	1.6%
IPI		
Low	32	50.00%
Low-intermediate	11	17.2%
High-intermediate	7	10.9%
High	12	18.8%
Undetermined	2	3.1%
Double-hit lymphoma		
Yes	2	3.1%
No	49	76.6%
Undetermined	13	20.3%
Biopsies		
Lymph node	26	40.6%
Extra-nodal	29	45.3%
Undetermined	9	14.1%
Relase status		
Non-relapse	41	64.1%
Relapse	20	31.2%
Unclassified	3	4.7%

international prognostic index, IPI; geminal center B-cell like, GCB.

Supplementary References

1. Ye, X., et al. A single-cell atlas of diffuse large B cell lymphoma. Cell Rep. **39**, 110713 (2022).
