# Supplementary material for: Spatially-resolved transcriptomics reveal macrophage heterogeneity and prognostic significance in diffuse large B-cell lymphoma
Source: Nat Commun. 2024 Mar 8;15:2113. doi: 10.1038/s41467-024-46220-z (PMC10923916; doi:10.1038/s41467-024-46220-z)
Supplement: Supplementary file 3 — Description of Additional Supplementary Files [file 41467_2024_46220_MOESM3_ESM.pdf]

Description of Additional Supplementary Files

Supplementary Data Legends

Supplementary Data 1. The DEGs of the comparsion between LZ and DZ

Supplementary Data 2. The gene lists of all MacroSigs with corresponding log2FC and adjusted P value

Supplementary Data 3. The DEGs of B cells between LZ and DZ

Supplementary Data 4. The cell numbers and AOI size in each region before quality control
