# Supplementary material for: Spatially-resolved transcriptomics reveal macrophage heterogeneity and prognostic significance in diffuse large B-cell lymphoma
Source: Nat Commun. 2024 Mar 8;15:2113. doi: 10.1038/s41467-024-46220-z (PMC10923916; doi:10.1038/s41467-024-46220-z)
Supplement: Supplementary file 8 — Reporting Summary [file 41467_2024_46220_MOESM8_ESM.pdf]

nature portfolio

Corresponding author(s): Claudio Tripodo and Anand D. Jeyasekharan

Last updated by author(s): Feb 6, 2024

Reporting Summary

Nature Portfolio wishes to improve the reproducibility of the work that we publish. This form provides structure for consistency and transparency in reporting. For further information on Nature Portfolio policies, see our Editorial Policies and the Editorial Policy Checklist.

Statistics

For all statistical analyses, confirm that the following items are present in the figure legend, table legend, main text, or Methods section.

n/a	Confirmed	
☐	☒	The exact sample size (*n*) for each experimental group/condition, given as a discrete number and unit of measurement
☐	☒	A statement on whether measurements were taken from distinct samples or whether the same sample was measured repeatedly
☐	☒	The statistical test(s) used AND whether they are one- or two-sided<br>*Only common tests should be described solely by name; describe more complex techniques in the Methods section.*
☐	☒	A description of all covariates tested
☐	☒	A description of any assumptions or corrections, such as tests of normality and adjustment for multiple comparisons
☐	☒	A full description of the statistical parameters including central tendency (e.g. means) or other basic estimates (e.g. regression coefficient) AND variation (e.g. standard deviation) or associated estimates of uncertainty (e.g. confidence intervals)
☐	☒	For null hypothesis testing, the test statistic (e.g. *F*, *t*, *r*) with confidence intervals, effect sizes, degrees of freedom and *P* value noted<br>*Give P values as exact values whenever suitable.*
☒	☐	For Bayesian analysis, information on the choice of priors and Markov chain Monte Carlo settings
☒	☐	For hierarchical and complex designs, identification of the appropriate level for tests and full reporting of outcomes
☒	☐	Estimates of effect sizes (e.g. Cohen's *d*, Pearson's *r*), indicating how they were calculated

Our web collection on statistics for biologists contains articles on many of the points above.

Software and code

Policy information about availability of computer code

Data collection	GeoMx DSP software 2.4.0.421(Nanostring); Nova seq 6000 (Illumina); HiSeq 3000 (Illumina); Vectra 2 System (PerkinElmer Inc., Waltham, MA, USA); inForm 2.6.0 (Akoya Biosciences).
Data analysis	R statistical software 4.3.0; GraphPad Prism software 9.5.0; Seurat 2.3.0; ClusterProfiler 3.12; Limma3.56.2; org.Hs.eg.db 3.18.0; ggplot2 3.4.4; survminer 0.4.9; FactoMinR 2.9; pheatmap 1.0.12.<br><br>The codes used for our manuscript are available in Zenodo (https://zenodo.org/doi/10.5281/zenodo.10511030).Source data are provided with this paper.

For manuscripts utilizing custom algorithms or software that are central to the research but not yet described in published literature, software must be made available to editors and reviewers. We strongly encourage code deposition in a community repository (e.g. GitHub). See the Nature Portfolio guidelines for submitting code & software for further information.

Data

Policy information about availability of data

All manuscripts must include a data availability statement. This statement should provide the following information, where applicable:

- Accession codes, unique identifiers, or web links for publicly available datasets
- A description of any restrictions on data availability
- For clinical datasets or third party data, please ensure that the statement adheres to our policy

The DSP RNA-seq raw data and processed data generated in this study have been deposited in GEO under accession code GSE232853 [https://www.ncbi.nlm.nih.gov/geo/query/acc.cgi?acc=GSE232853]. DLBCL expression array datasets of Lenz et al 64 (GSE10846, n = 420, [https://www.ncbi.nlm.nih.gov/geo/query/acc.cgi?acc=GSE10846]), Visco et al 62 (GSE31312, n = 498, [https://www.ncbi.nlm.nih.gov/geo/query/acc.cgi?acc=gse31312]), Dubois et al 65 (GSE87371, n = 223, [https://www.ncbi.nlm.nih.gov/geo/query/acc.cgi?acc=GSE87371]), Chapuy et al 66 (GSE98588, n = 137, [https://www.ncbi.nlm.nih.gov/geo/query/acc.cgi?acc=GSE98588]), Sha et al 67 (GSE117556, n = 913, [https://www.ncbi.nlm.nih.gov/geo/query/acc.cgi?acc=GSE117556]), and Lacy et al 39 (GSE181063, n = 1,149, [https://www.ncbi.nlm.nih.gov/geo/query/acc.cgi?acc=GSE181063]) were obtained from Gene Expression Omnibus. DLBCL RNA-seq dataset, Reddy et al 61 (n = 773) , was obtained through The European Genome-phenome Archive (EGA) at the European Bioinformatics Institute, Study ID: EGAS00001002606 [https://ega-archive.org/studies/EGAS00001002606]. This is a restricted access dataset that can be obtained through a data access request application as instructed on the EGA portal (https://ega-archive.org/). Schmitz et al 68 (n = 481) was obtained from the National Institutes of Health (NIH) database of Genotypes and Phenotypes (dbGaP), accession number: phs001444.v2.p1 [https://www.ncbi.nlm.nih.gov/projects/gap/cgi-bin/study.cgi?study_id=phs001444.v2.p1]. This is a restricted access dataset that can be obtained through a data access request application as instructed on the dbGaP portal (https://www.ncbi.nlm.nih.gov/gap/). Single-cell RNA sequencing (scRNA-seq) datasets of tonsil were obtained from HCATonsilData 59 [https://bioconductor.org/packages/release/data/experiment/html/HCATonsilData.html]. The scRNA-seq dataset of DLBCL from Ye et al 69 (n = 17) is available at the CNGB Sequence Archive (CNSA) of the China National GeneBank DataBase (CNGBdb) under accession number CNP0001940 [https://db.cngb.org/search/project/CNP0001940/]. This is a restricted access dataset that can be obtained through a data access request application as instructed on the CNGBdb portal (https://db.cngb.org/). The integrative scRNAseq dataset for human monocytes and macrophages (MoMac-VERSE; from 41 scRNA-seq datasets comprising 13 healthy and pathological tissues), was obtained from Mulder et al 33 [https://macroverse.gustaveroussy.fr/2021_MoMac_VERSE/]. The above-mentioned n in this section refers to patient numbers. Source data are provided with this paper.

Research involving human participants, their data, or biological material

Policy information about studies with human participants or human data. See also policy information about sex, gender (identity/presentation), and sexual orientation and race, ethnicity and racism.

Reporting on sex and gender	Sex and gender were not considered in study design; proportion of male and female samples are indicated.
Reporting on race, ethnicity, or other socially relevant groupings	Race, ethnicity, or other socially relevant groupings were not considered in our study design or reported in the manuscript.
Population characteristics	DLBCL patients from NUH cohort: Age: ≤60y 40.6%, ?60y 59.4%; Sex: Male 5.6%, Female 34.4%; Cell of origin: Non-GCB 50.0%, GCB 48.4%; Undetermined 1.6%; IPI: Low 50.00%, Low-intermediate 17.2%, High-intermediate 10.9%, High 18.8%, Undetermined 3.1%; Double-hit lymphoma: Yes 3.1%, No 76.6%, Undetermined 20.3%; Relase status: Non-relapse 64.1%, Relapse 31.2%, Unclassified 34.7%.
Recruitment	Patients were not directly recruited to this study: suitable biological samples were retrospectively selected for this exploratory analysis based on availability of complete corresponding clinical information and age of the sample (sample was required to have been collected post-2010).
Ethics oversight	All biopsy samples were obtained from the Department of Pathology, National University Hospital, in accordance with the ethical guidelines of the domain specific review board (DSRB) approved protocol 2015/00176.

Note that full information on the approval of the study protocol must also be provided in the manuscript.

Field-specific reporting

Please select the one below that is the best fit for your research. If you are not sure, read the appropriate sections before making your selection.

☒ Life sciences ☐ Behavioural & social sciences ☐ Ecological, evolutionary & environmental sciences

For a reference copy of the document with all sections, see nature.com/documents/nr-reporting-summary-flat.pdf

Life sciences study design

All studies must disclose on these points even when the disclosure is negative.

Sample size	Group 1 comprised of two tissue microarrays (TMAs) of de novo DLBCL samples derived from pre-treatment biopsies of 64 patients between 2010 and 2017 subsequently treated with 6x R-CHOP with a follow-up time more than three years (for relapsed patients, the follow-up time are at least 1 year), at the National University Hospital in Singapore. 23 patients had duplicate cores between both TMAs, meaning a total of 87 biopsies were profiled. Patient details and characteristics from the aforementioned cohorts are summarized in Supplementary Table 5.

Group 2 (non-malignant reactive lymphoid tissue samples) comprised of a TMA containing 12 tonsil samples and 12 tonsil whole-slide samples, obtained from patients with tonsillectomies at the National University Hospital for non-cancer indications. An additional DLBCL TMA from the CMMC cohort (n = 86) with OS more than 6 months, was used as a validation cohort for quantitative immunofluorescence analyses. A sample size calculation was not performed: biological samples were selected for this exploratory analysis based on availability of complete corresponding clinical information and age of the sample (sample was required to have been collected post-2010).

Data exclusions	No data was excluded from the analysis.
Replication	The replication number was indicated in the main text and Figures of this study.
Randomization	Patients were not directly recruited to this study: suitable biological samples were retrospectively selected for this exploratory analysis based on availability of complete corresponding clinical information and age of the sample (sample was required to have been collected post-2010).
Blinding	Blinding is not relevant as this study is an exploratory analysis with no preconceived notion of results obtained.

Reporting for specific materials, systems and methods

We require information from authors about some types of materials, experimental systems and methods used in many studies. Here, indicate whether each material, system or method listed is relevant to your study. If you are not sure if a list item applies to your research, read the appropriate section before selecting a response.

Materials & experimental systems

n/a	Involved in the study	
☐	☒	Antibodies
☒	☐	Eukaryotic cell lines
☒	☐	Palaeontology and archaeology
☒	☐	Animals and other organisms
☒	☐	Clinical data
☒	☐	Dual use research of concern
☒	☐	Plants

Methods

n/a	Involved in the study	
☒	☐	ChIP-seq
☒	☐	Flow cytometry
☒	☐	MRI-based neuroimaging

Antibodies

Antibodies used

Macrophage marker CD68 (sc-20060 AF594, 1:100, KP1, Santa Cruz biotechnology, Texas, USA)
T-cell marker CD3 (Dako, A0452, 1:200, Polyclonal, California, USA)
B-cell marker CD20 (Novus Biologicals, NBP2-47840 AF647, 1:100, IGEL/773, Colorado, USA)
Follicular dendritic cell marker nerve growth factor receptor (NGFR, Ab52987, 1: 800, EP1039Y, Abcam, Cambridge, UK)
Donkey anti-Rabbit IgG (H+L) Highly Cross-Adsorbed Secondary Antibody, Alexa Fluor™ Plus 555, 1:400, Thermo Fisher Scientific, USA

Validation

All antibodies are from commercial companies and are well validated by manufacturer and widely used in researches. Their validation data are available on the manufacturer's websites:
Macrophage marker CD68 (sc-20060 AF594)
https://www.scbt.com/p/cd68-antibody-kp1

T-cell marker CD3 (Dako, A0452)
https://www.agilent.com/en/product/immunohistochemistry/antibodies-controls/primary-antibodies/cd3-(concentrate)-76133

B-cell marker CD20 (Novus Biologicals, NBP2-47840 AF647)
https://www.novusbio.com/products/cd20-antibody-igel-773_nbp2-47840af647

Follicular dendritic cell marker nerve growth factor receptor (NGFR, Ab52987)
https://www.abcam.com/en-hk/products/primary-antibodies/p75-ngf-receptor-antibody-ep1039y-ab52987

Donkey anti-Rabbit IgG (H+L) Highly Cross-Adsorbed Secondary Antibody, Alexa Fluor™ Plus 555,A32794
https://www.thermofisher.com/antibody/product/Donkey-anti-Rabbit-IgG-H-L-Highly-Cross-Adsorbed-Secondary-Antibody-Polyclonal/A32794
